# Predictive Accuracy of Intraocular Lens Formulas Calculated by Biometers with Multiple Refractive Indices According to Axial Length

**DOI:** 10.3390/jcm13226815

**Published:** 2024-11-13

**Authors:** Yeo Kyoung Won, Young-Sik Yoo, Hee-jee Yun, Tae-Young Chung, Dong Hui Lim

**Affiliations:** 1 Department of Ophthalmology, Samsung Medical Center, Sungkyunkwan University School of Medicine, Seoul 06351, Republic of Korea; 2 IU Eye Clinic, Seoul 05606, Republic of Korea; 3 Renew Seoul Eye Center, Seoul 06181, Republic of Korea; 4 Department of Medical Device Management and Research, Samsung Advanced Institute for Health Sciences and Technology, Sungkyunkwan University, Seoul 06351, Republic of Korea

**Keywords:** intraocular lens, intraocular lens power calculation formula, swept-source optical coherence tomography, optical biometry, multiple refractive indices, specific refractive index

## Abstract

**Background/Objectives:** This study aimed to analyze the accuracy of the SRK/T, Haigis, Barrett Universal II (BUII), Kane, and EVO intraocular lens (IOL) formulas for normal and long axial length (AL) groups using an ARGOS (Alcon, Fort Worth, TX, USA), which uses the specific refractive indices. **Methods:** We performed a review of patients who underwent uneventful cataract surgery with the implantation of an Acrysof IQ SN60WF IOL (Alcon, Inc.) between January 2020 and December 2021. Biometry was obtained with the ARGOS; patients were separated into subgroups based on AL as follows: normal (22.0 ≤ AL < 26.0 mm) and long (AL ≥ 26.0 mm). Outcomes included the mean error (ME), the mean absolute error (MAE), the median absolute error (MedAE), and the proportion of eyes within ±0.25, ±0.50, ±0.75, and ±1.00 diopters (D) of the prediction error. **Results:** A total of 191 eyes of 191 patients were included: 162 eyes of 162 patients in the normal AL group and 29 eyes of 29 patients in the long AL group. The EVO formula was the most accurate for the normal AL group, which had the lowest MAE and MedAE. The MAE and MedAE of EVO were the lowest in the long AL group; EVO showed the highest percentage of eyes within ±0.25, ±0.75, and 1.00 D compared with other formulas. **Conclusions:** When using an ARGOS, the EVO formula had the lowest MAE and the highest proportion of eyes within ±1.00 D of the predicted target in both the normal and high myopia groups.

## 1. Introduction

The accurate prediction of intraocular lens (IOL) power before cataract surgery is important for achieving good visual outcomes after cataract surgery. The prediction of postoperative refractive outcomes has steadily improved with newly developed ocular biometry devices and more recent IOL power calculation formulas [1,2,3]. Accurate IOL power requires precise ocular biometrics, in which axial length (AL) plays a major role [4,5]. A new type of ocular biometry device using the principle of swept-source optical coherence tomography (SS-OCT) has been developed in recent years [5]. Since SS-OCT-based devices use a light source with longer wavelengths and have superior medium permeability compared to a partial coherence interferometry (PCI)-based device, they may provide a more accurate AL [5,6]. An ARGOS (Alcon, Fort Worth, TX, USA) is a device that considers the specific refractive index of each segment of the eye (cornea, anterior chamber, lens, and vitreous), rather than a single, equivalent, composite refractive index [7]. As such, the AL calculation is appropriately adjusted and more accurate by considering the difference in refractive index [8,9]. This increase in predictive accuracy is more evident in short and long eyes [7].

While predicting refractive error after cataract surgery was primarily based on ocular biometry measurements in the past, advances in measurement accuracy have revealed that the main cause of errors in refractive power prediction after surgery involves the effective lens position (ELP). In 2008, Norrby estimated that 35.5% of IOL power calculation errors were caused by an ELP prediction error [10]. ELP prediction is an important variable in the latest IOL power calculation formula. Unlike earlier formulas, the latest IOL power calculation formula predicts ELP using various variables. For example, the Haigis formula estimates ELP from the AL and anterior chamber depth (ACD) [11,12], while the Barrett Universal II (BUII) formula includes parameters such as ACD and lens thickness [13,14].

In the cases of eyes with a long AL, it remains challenging to estimate the ELP [15]. Moreover, existing studies indicate that if a measurement error of 1 mm in the AL occurs, the power of the IOL can change by approximately 2.7 diopters (D) [16]. Thus, especially in highly myopic eyes, a more accurate formula and measurement method considering the specific refractive index for each segment will be needed.

The fourth-generation formulas, including the Haigis and the latest formulas such as BUII, Kane, and EVO, are now used for calculating the power of IOLs; a comparison of the accuracy of these formulas using a biometer with a specific refractive index in short and long eyes has been reported [7]. However, to our knowledge, no study has compared them in normal and long eyes with these formulas using the ARGOS.

The aim of this study was to analyze the accuracy of the SRK/T, Haigis, BUII, Kane, and EVO formulas in normal and long AL groups using the ARGOS.

## 2. Materials and Methods

### 2.1. Subjects

We performed a retrospective review of 191 patients who underwent cataract surgery with the implantation of an Acrysof IQ SN60WF IOL (Alcon Laboratories, Inc., Fort Worth, TX, USA) between January 2020 and December 2021 at the Samsung Medical Center, Republic of Korea. The study protocol was approved by the Institutional Review Board of Samsung Medical Center (IRB #2022-03-092) and was performed in compliance with the tenets of the Declaration of Helsinki.

The inclusion criterion was uneventful cataract surgery, with in-the-bag insertion of an Acrysof IQ SN60WF IOL performed by two surgeons (T-Y.C. and D.H.L.). AL, ACD, and keratometry (K) in the eyes of all patients were measured preoperatively using an ARGOS (Alcon, Fort Worth, TX, USA), a device based on SS-OCT. The exclusion criteria were any corneal or ocular disease, prior corneal or intraocular surgery, complicated cataract surgery, postoperative corrected distance visual acuity (CDVA) < 20/40, or postoperative complications. If both eyes met the inclusion criteria, only one eye was randomly selected and included to avoid the compounding of data. Manifest refraction was performed at 1 month postoperatively. Patients with 22.0 mm ≤ AL < 26.0 mm and AL ≥ 26.0 mm were divided into normal (Group 1) and long (Group 2) AL groups, respectively.

### 2.2. Surgical Technique

All surgical procedures were performed using a standardized, suture-free phacoemulsification technique, with a 2.75 mm clear corneal incision in the temporal region under topical anesthesia by one of two experienced surgeons (T-Y.C. and D.H.L.). Postoperative gatifloxacin (Gatiflo^®^; Handok, Seoul, Republic of Korea) and fluorometholone 0.1% eye drops (Flumetholon^®^; Santen, Seoul, Republic of Korea) were administered four times per day for 1 month.

### 2.3. Patient Evaluation

Using the software built into the ARGOS, the IOL power was calculated using the SRK/T, Haigis, and BUII formulas. Moreover, we evaluated the performance of online formula calculators, including Kane (version 1.0, available at: http://iolformula.com (accessed on 22 June 2022)) and EVO (version 2.0, available at: https://www.evoiolcalculator.com/calculator.aspx (accessed on 22 June 2022)). We used the ARGOS version of the EVO formula, which is available on its own website.

The accuracy of the formulas was evaluated using the following steps: First, each prediction error (PE) was back-calculated as the difference between the actual postoperative spherical equivalent (SE) and the formula-predicted SE based on the implanted IOL power. The mean error (ME), mean of all PEs for each formula, and standard deviation (SD) of the PE were reported. We individually optimized the A constant for SRK/T, BUII, Kane, and EVO and a_o_ for Haigis to zero ME using a specific computer programming language obtained with Python software (version 3.12, Python Software Foundation, available at http://www.python.org (accessed on 22 June 2022)). Second, to compare the predictive accuracy of IOL power formulas, we determined the mean absolute error (MAE) and the median absolute error (MedAE), which are the mean and median of the absolute value of each PE, respectively. We also determined the percentages of eyes within ±0.25 D, ±0.50 D, ±0.75 D, and ±1.0 D of PE for each formula.

### 2.4. Statistical Analysis

Statistical analyses were performed using R 4.1.3 (Vienna, Austria; http://www.R-project.org/ (accessed on 22 June 2022)). Means and SDs or percentages were used to report the accuracy of the formulas. The normality of the data was tested using the Kolmogorov–Smirnov test. To test whether the ME was significantly different from zero, a 1-sample *t*-test was used because the data were normally distributed. Differences in the PE and MAE between the formulas were assessed using the Friedman test. In the event of a significant result, a post hoc analysis was performed using the Wilcoxon signed-rank test with the Bonferroni correction.

The Cochran Q test was performed to compare the percentages of eyes with PE within ±0.25 D, ±0.50 D, ±0.75 D, and ±1.0 D of the target refraction. Statistical significance was set at *p* < 0.05.

## 3. Results

In total, 191 eyes of 191 patients were included in this retrospective study and divided into two groups according to AL: 162 eyes of 162 patients (Group 1) and 29 eyes of 29 patients (Group 2). The baseline characteristics and preoperative measurements of the patients are presented in Table 1. Significant differences between the groups were found only in AL (*p* < 0.001). No significant differences were observed in age, sex, laterality, ACD, or K (*p* > 0.05).

**Figure 1:**
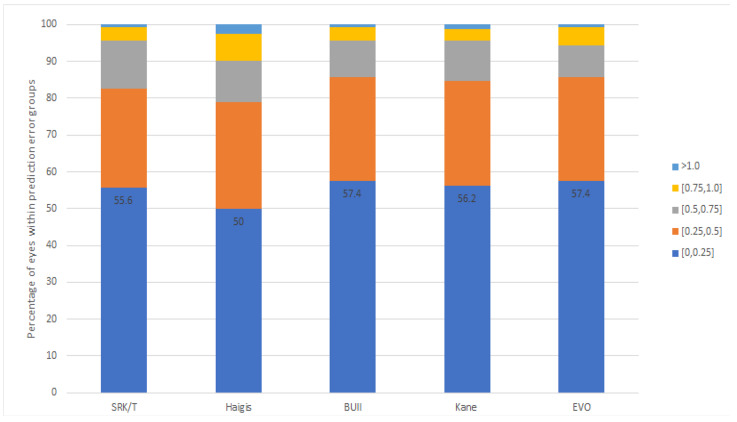
Stacked histogram comparing the percentage of cases within a given diopter range of predicted refractive outcomes in the normal AL (Group 1).

Table 2 shows refractive errors calculated using the five IOL formulas (SRK/T, Haigis, BUII, Kane, and EVO) in normal AL (Group 1). The MEs of all constant-optimized formulas were not different from zero. The MAEs ranged from 0.26 D to 0.33 D, while the MedAEs ranged from 0.19 D to 0.25 D. The most accurate formula was the EVO formula, which showed the lowest MAE (0.26 D) and MedAE (0.19 D). There was a statistically significant difference in MAE between formulas (*p* = 0.043, Friedman test). However, through post hoc analysis, the differences were statistically significant only between EVO and Haigis and between BUII and Haigis (*p* = 0.0019 and 0.002, respectively). Following the EVO formula, BUII showed the second-lowest MedAE (0.20 D) and MAE (0.27 D). The percentage of eyes that had a prediction error within ±0.25 D, ±0.5 D, ±0.75 D, and ±1.0 D for each formula in normal AL (Group 1) is shown in Figure 1 and Table 2. There were no statistically significant differences in the percentage within ±0.25 D, ±0.5 D, and ±0.75 D between the five IOL formulas (*p* > 0.05). However, in terms of accuracy within ±0.25 D and ±0.5 D, the EVO and BUII formulas tended to show the highest percentage of eyes between the formulas, followed by Kane. Within ±1.0 D, the EVO, BUII, and SRK/T all showed the highest accuracy at the same rate.

**Figure 2:**
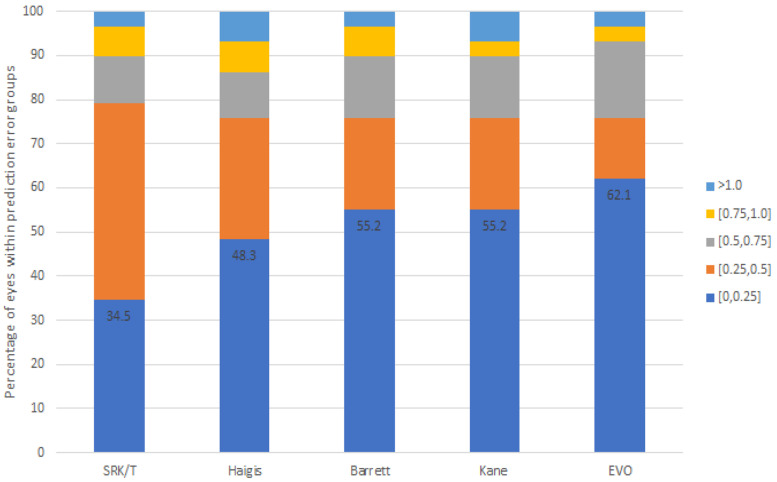
Stacked histogram comparing the percentage of cases within a given diopter range of predicted refractive outcomes in the long AL group (Group 2).

The refractive errors and percentages of eyes that had a prediction error within ±0.25 D, ±0.5 D, ±0.75 D, and ± 1.0 D in long AL (Group 2) are summarized in Table 3 and Figure 2. In all formulas, the MEs did not differ from zero. For long eyes, the MAEs ranged from 0.30 D to 0.36 D, while the MedAEs ranged from 0.18 D to 0.27 D. There was no statistically significant difference in the MAE between the formulas (*p* = 0.294, Friedman test). Similarly to the normal AL group, the MAE (0.30 D) and MedAE (0.18 D) of the EVO formula were the lowest in the long AL group. In addition, there was a significant difference in the percentages within ±0.25 D between the formulas (*p* = 0.037, Cochran Q test). The SRK/T formula achieved the highest accuracy within ±0.5 D, with a rate of 79.3%, while the others had an accuracy of 75.9%. However, the EVO formula demonstrated a considerably higher percentage of eyes within the remaining prediction error targets compared to the other formulas. The percentage within ±0.25 D, ±0.75 D, and ±1.0 D of the EVO formulas were 62.1%, 93.1%, and 96.6%, respectively.

Patients were divided into two sub-groups based on the axial length: normal AL group (Group 1, 22.0 mm ≤ AL < 26.0 mm) and long AL group (Group 2, AL ≥ 26.0 mm).

## 4. Discussion

The main potential causes of prediction error in IOL power calculation include the accuracy of the AL measurement and the IOL power calculation formula used [1,2,3,17]. In particular, IOL power calculation for highly myopic eyes remains challenging, often resulting in unexpected hyperopic outcomes [17]. In this study, we obtained preoperative biometry based on an ARGOS, which can accurately measure AL using multiple refractive indices, and compared the accuracy of the latest IOL power calculation formulas in patients with normal and long ALs. Among these formulas, the EVO formula provided the best predictability in both normal and high myopia when using the ARGOS.

The ARGOS is an optical biometer based on SS-OCT that considers the specific refractive index rather than the composite refractive index; it provides more accurate AL values by considering the difference in the refractive index of media, such as the cornea and vitreous body. When light reaches the retinal pigment epithelium (RPE), it passes through the cornea, aqueous humor, lens, and vitreous body; all of which have different refractive indices. When calculating the length using a single refractive index, the composite refractive index was set to 1.3375, which was greater than the refractive index of the vitreous body (1.336). Thus, when measuring long AL using the composite method, the length of the vitreous body, which light must pass through to reach the RPE, is large. Therefore, it is bound to be longer than the actual AL. Several studies subsequently demonstrated that the ARGOS displays a tendency to measure long eyes shorter and closer to the actual AL, in comparison to optical biometers using composite refractive index, such as the IOL Master 700 (Carl Zeiss Meditex, Jena, Germany), the Lenstar LS900 (Haag-Streit AG, Koeniz, Switzerland), the OA-2000 (Tomey, Nagoya, Japan), and the Pentacam AXL (Oculus, Weizlar, Germany) [18,19,20,21]. To overcome this bias present in existing optical biometers, the Wang–Koch adjustment was used for the formulas (SRK/T and Holladay I) in patients with long AL [3,15,22,23,24,25].

Several recent studies showed that the refractive accuracy of IOL power formulas was higher in eyes measured with a specific refractive index than in eyes measured with a composite refractive index [5,7,8,9,23]. Shammas et al. showed that the absolute error was lower for the specific refractive index group in 53.8% to 62.2% of cases [26]. Accordingly, as traditional IOL power calculation formulas were developed for the conventional biometry, newer IOL formulas such as the EVO, PEARL-DGS, and Barrett True AL (BTAL) have currently been introduced for biometry that calculates the segmental index [7,27]. However, the software installed in the ARGOS device also supports the use of traditional IOL power formulas. Additionally, Omoto et al. reported that the ARGOS uses the same optimized IOL constants as the Zeiss IOL Master, since IOL constants specialized for ARGOS are available at present, and the manufacturer recommends their use [21]. Yang et al. reported that the postoperative MAE of Haigis was lower for the ARGOS than for existing IOL Masters [5].

The EVO formula showed the highest accuracy in both the long and normal AL groups. Similarly, the previous study by Shammas et al. also demonstrated that EVO was one of the formulas with the lowest MAE (0.29 D) in the group with AL > 24.5 mm when using the ARGOS [7]. However, in the group with AL > 25.0 mm, BUII and Kane had a lower MAE of 0.27 D, followed by EVO (0.28 D) [7]. Since a difference in the definition of high myopia was noted, it is difficult to make a direct comparison from our study. However, the good refractive outcomes of the EVO formula are comparable with our results. As shown on its homepage (EVO Version 2.0), the EVO formula can be adjusted for measurements based on the ARGOS biometer, which may lead to higher accuracy as compared to other formulas in this study, although the A constant between the traditional optical biometry and the ARGOS may be interchangeable. In the future, it will be necessary to compare the accuracy with the PEARL-DGS or BTAL formulas as well.

To date, most studies on the accuracy of recent IOL formulas, including EVO, have been based on conventional optical biometers, such as PCI- or SS-OCT-based IOL Masters. Among normal eyes, EVO, BUII, and Kane showed the highest accuracy when using different biometers [28,29,30]. EVO was also the most accurate in long eyes [24,29,31,32]. Even in extremely long AL ≥ 30 mm, EVO still showed the highest accuracy [33]. Regardless of the type of optical biometer used, it is evident that EVO relatively exhibited the highest accuracy when compared to other formulas. Meanwhile, Omoto et al. showed that the Kane formula had excellent prediction accuracy in long eyes [34]. Darcy et al. also reported its superiority, which had the lowest MAE as compared with other modern formulas in long eyes [35]. However, in this study, the results of the Kane formula were not as good as expected; the MAEs were higher in the long AL group compared to in other studies. This may have been likely due to the difference in the measurement principles of the optical biometer and the relatively smaller sample size in our study. Therefore, for a more accurate comparison with other studies, further research with a larger sample size is needed.

This study had several limitations. First, we included a relatively small number of eyes, particularly in the long AL group. Only one eye with extremely high myopia, defined as AL > 30 mm, was included in this study, thereby suggesting the need for a larger sample size in future research. It may also be necessary to analyze groups with extremely high myopia and high myopia separately. In addition, we did not account for the myopic cone or effective diopter value of high myopia in this study. The primary focus was on axial length as the key parameter influencing the accuracy of IOL power formulas. However, as the changes in refractive power and the presence of a myopic cone may potentially impact the measurements and outcomes, further research that includes those factors will be necessary for a more comprehensive evaluation. Second, since Yeo and Barrett found that slightly better results were achieved without ACD measurements using PCI, we plan to include the outcomes of the EVO or BUII formulas without ACD measurements using the ARGOS in a future study [36]. Furthermore, a comparison of accuracy with other recently introduced IOL formulas, such as the Hill-RBF, K6, BTAL, PEARL-DGS, and Ladas Super formulas, would be needed. However, this study remains meaningful, as it is the first to demonstrate the superiority of the EVO formula in both normal and high myopic eyes using the ARGOS.

In conclusion, when using the ARGOS, which measures AL more accurately by considering the specific refractive index of each eye segment based on SS-OCT, the EVO formula had the lowest MAE and MedAE, along with the highest proportion of eyes within ±0.25 D, ±0.50 D, ±0.75 D, and ±1.00 D of the predicted target in both the normal and long AL groups. These findings suggest that the EVO formula offers excellent accuracy, making it a reliable choice for IOL power prediction over a wide range of axial lengths, particularly when employing advanced biometry that uses the specific refractive indices.

## Figures and Tables

**Table 1:** Demographics and baseline data of patients.

	Normal AL (Group 1)	Long AL (Group 2)	*p* Value *
Eyes (number)	162	29	
Age (years)	71.23 ± 8.87	62.14 ± 12.28	0.309
Laterality (right–left)	79:83	13:16	0.696 ^†^
Female [number (%)]	70 (43.2%)	14 (48.3%)	0.125 ^†^
AL (mm)	23.60 ± 0.80	27.72 ± 1.37	<0.001
ACD (mm)	3.19 ± 0.37	3.62 ± 0.40	0.331
Flat K (D)	43.57 ± 1.43	43.46 ± 1.38	0.906
Steep K (D)	44.45 ± 1.46	44.46 ± 1.44	0.934
Mean K (D)	44.01 ± 1.41	43.96 ± 1.38	0.907

* Independent *t*-test. ^†^ Chi-square test; mean ± standard deviation; AL = axial length; ACD = anterior chamber depth; K = keratometry.

**Table 2:** Predictive accuracy in the normal AL group (Group 1).

	PE (D)	*p* Value *	MAE	MedAE	IQR	PE ± 0.25 D	PE ± 0.5 D	PE ± 0.75 D	PE ± 1.0 D	Range (D)
SRK-T	0.00 ± 0.36	1.000	0.28	0.22	0.45	55.6%	82.7%	95.7%	99.4%	−0.85 to 1.30
Haigis	−0.00 ± 0.42	1.000	0.33	0.25	0.53	50.0%	79.0%	90.1%	97.5%	−1.14 to 1.14
Barrett	0.00 ± 0.35	1.000	0.27 ^§^	0.20	0.42	57.4%	85.8%	95.7%	99.4%	−0.98 to 1.18
Kane	0.00 ± 0.37	1.000	0.28	0.22	0.46	56.2%	84.6%	95.7%	98.8%	−1.04 to 1.18
EVO	0.00 ± 0.35	1.000	0.26 ^‡^	0.19	0.40	57.4%	85.8%	94.4%	99.4%	−0.92 to 1.21
*p* value ^†^	0.573		0.043			0.231	0.068	0.106	0.575	

AL = axial length; PE = prediction error; MAE = mean absolute error; MedAE = median absolute error; IQR = interquartile range; D = diopter; Barrett = Barrett Universal II. * Difference from zero by the one-sample T test. ^†^ Difference among the arithmetic (PE) and absolute (MedAE and MAE) PEs of the five formulas (Friedman test) and among the percentages of eyes within each PE (Cochran Q test). ^‡,§^ Significant difference in EVO and Barrett from Haigis in post hoc analysis, respectively, calculated using pairwise Wilcoxon signed-rank test with Bonferroni correction for the values. Patients with 22.0 mm ≤ AL < 26.0 mm were included in the normal AL group.

**Table 3:** Predictive accuracy in the long AL group (Group 2).

	PE (D)	*p* Value *	MAE	MedAE	IQR	PE ± 0.25 D	PE ± 0.5 D	PE ± 0.75 D	PE ± 1.0 D	Range (D)
SRK-T	0.00 ± 0.43	1.000	0.36	0.26	0.515	34.5%	79.3%	89.7%	96.6%	−0.76 to 1.06
Haigis	0.00 ± 0.48	1.000	0.34	0.27	0.39	48.3%	75.9%	86.2%	93.1%	−1.00 to 1.34
Barrett	−0.00 ± 0.47	1.000	0.34	0.24	0.395	55.2%	75.9%	89.7%	96.6%	−0.86 to 1.53
Kane	−0.00 ± 0.48	1.000	0.35	0.21	0.385	55.2%	75.9%	89.7%	93.1%	−0.83 to 1.28
EVO	0.00 ± 0.41	1.000	0.30	0.18	0.36	62.1%	75.9%	93.1%	96.6%	−0.94 to 1.09
*p* value ^†^	0.594		0.294			0.037	0.973	0.287	0.788	

AL = axial length; PE = prediction error; MAE = mean absolute error; MedAE = median absolute error; IQR = interquartile range; D = diopter; Barrett = Barrett Universal II. * Difference from zero by the one-sample T test. ^†^ Difference among the arithmetic (PE) and absolute (MedAE and MAE) PEs of the five formulas (Friedman test) and among the percentages of eyes within each PE (Cochran Q test). Patients with AL ≥ 26.0 mm were included in the long AL group.

## Data Availability

The data presented in this study are available on request from the corresponding author.

## Abbreviations

ACD	anterior chamber depth
AL	axial length
BUII	Barrett Universal II
D	diopter
ELP	effective lens position
IOL	intraocular lens
PCI	partial coherence interferometry
IRB	institutional review board
RPE	retinal pigment epithelium
SS-OCT	swept-source optical coherence tomography
BTAL	Barrett True AL
